# Dental Society of New York

**Published:** 1872-04

**Authors:** 


### PROCEEDINGS OF SOCIETIES.

### The Dental Society of the State of New York—Special Meeting.

At the request of the several district societies of the State, the Executive Board called a meeting of the above Society, at the Mott Memorial Rooms, in the city of New York, on Tuesday, February 13th, 1872, for the purpose of uniting in a plan to contest the validity of the so-called Cummings Patent, and prevent the extension of the Goodyear Rubber Patent.

The dentists of New York and Brooklyn at a mass meeting appointed a committee of ten to watch and conduct, on the part of the dentists, the defense in what is known as the Gardner suit. The committee reported what had been done by them, and suggested to the Society what seemed to them the best course to pursue.

A resolution was passed requesting the several District Societies to appoint a committee to act in concert with the committee now appointed by the dentists of New York and Brooklyn, for the purpose of raising money to pay counsel fees in testing the validity of the so-called Cummings Patent and the extension of the Goodyear Patent.

A committee of five were appointed to circulate a petition to Congress against the extension of the Goodyear Patent.

The following resolution was unanimously passed:

*Resolved*, That we have heard with satisfaction of the action taken by the committee of ten appointed by the dentists of New York and Brooklyn in the suit between the Goodyear Dental Vulcanite Company and Dr. Gardner, and that we heartily approve the course pursued by them, and pledge ourselves to do all in our power to assist them.

CHARLES BARNES, Secretary.

### Executive Committee Meeting.

At a meeting of the Executive Committee of New York and Brooklyn, March 21, 1872, to hear the report of the delegates to Washington, the following resolutions were adopted, and it was desired that copies should be sent to the dental journals for publication, as a tribute eminently due to the gentlemen mentioned:

*Resolved*, That the energy, ability, and integrity which have been shown by our counsel, Col. John A. Foster and Samuel J. Glassey, Esq., in the preparation and presentation of the case of B. E. Gardner against the Cummings Patent—just argued in the United States Supreme Court at Washington—entitle them to this recognition of their services by the committee, and merit this public acknowledgment of their untiring labor, and skillful management of the case; also

*Resolved*, That the committee gladly recognize their obligations to the gentlemen of the Baltimore and Washington committees, and to their counsel, Orville Horwibs, Esq., for the efficient support they have so promptly and cheerfully given to the measures which it is hoped will result in our speedy and final success. C. A. Woodward, Chairman.

W. A. Bronson, Secretary.

New York, March 23, 1872.

